# Supplementary material for: Strong protective effect of the APOL1 p.N264K variant against G2-associated focal segmental glomerulosclerosis and kidney disease
Source: Nat Commun. 2023 Nov 30;14:7836. doi: 10.1038/s41467-023-43020-9 (PMC10689833; doi:10.1038/s41467-023-43020-9)
Supplement: Supplementary file 1 — Supplementary Information [file 41467_2023_43020_MOESM1_ESM.pdf]

Supplementary Information

Strong protective effect of the *APOL1* p.N264K variant against G2-associated FSGS and kidney disease

SUPPLEMENTARY FIGURES

Supplementary Figure 1. Principal component analysis of FSGS cohorts.

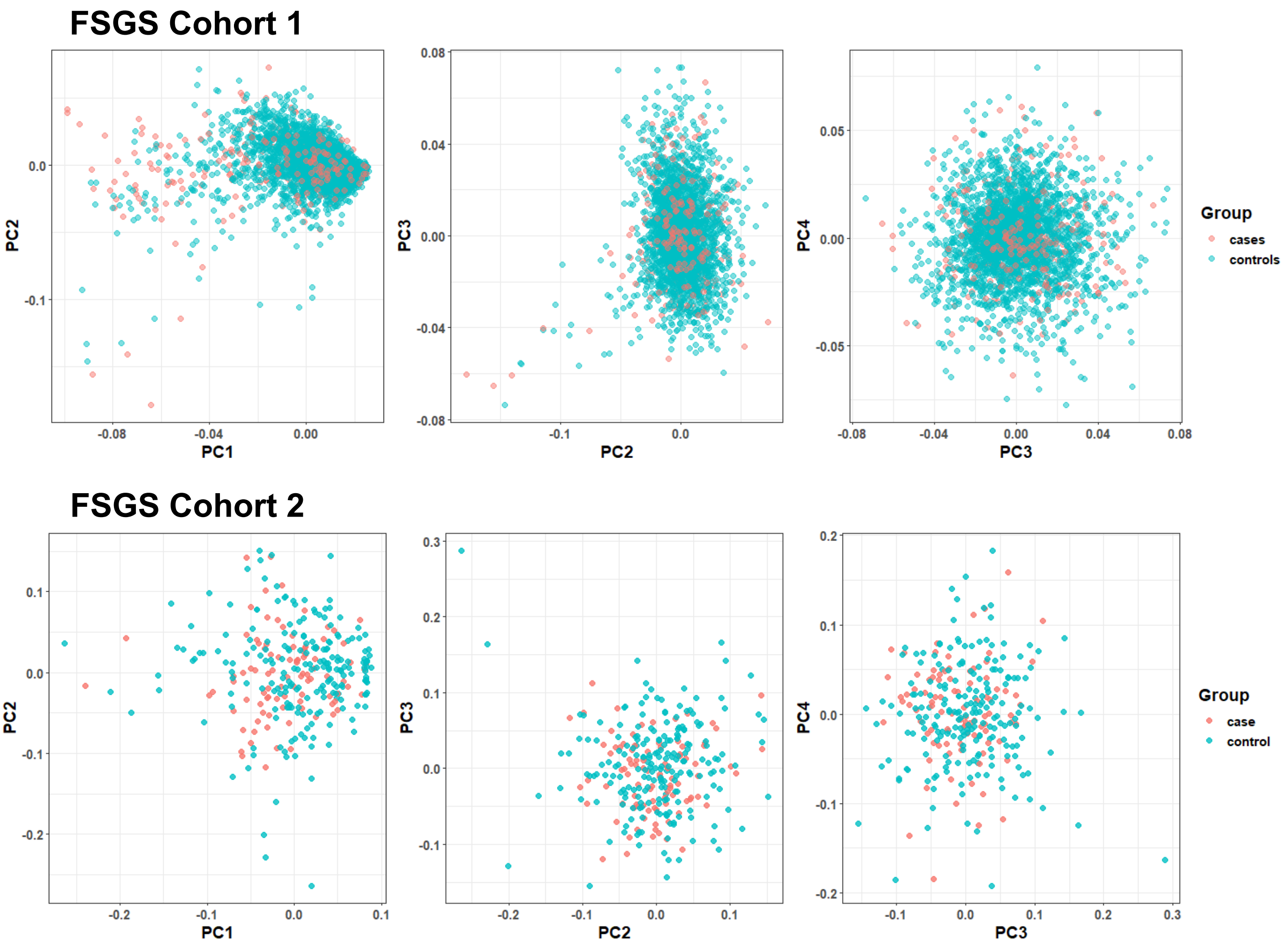

The figure shows plots for the first three principal components for the FSGS cohort 1 based on 279,502 pruned SNPs and for the FSGS cohort 2 based on 219,146 pruned SNPs. PCA shows well-matched case-control data for both cohorts.

PC=principal component.

Supplementary Figure 2. *APOL1* p.N264K association analysis in FSGS cohorts 1 and 2.

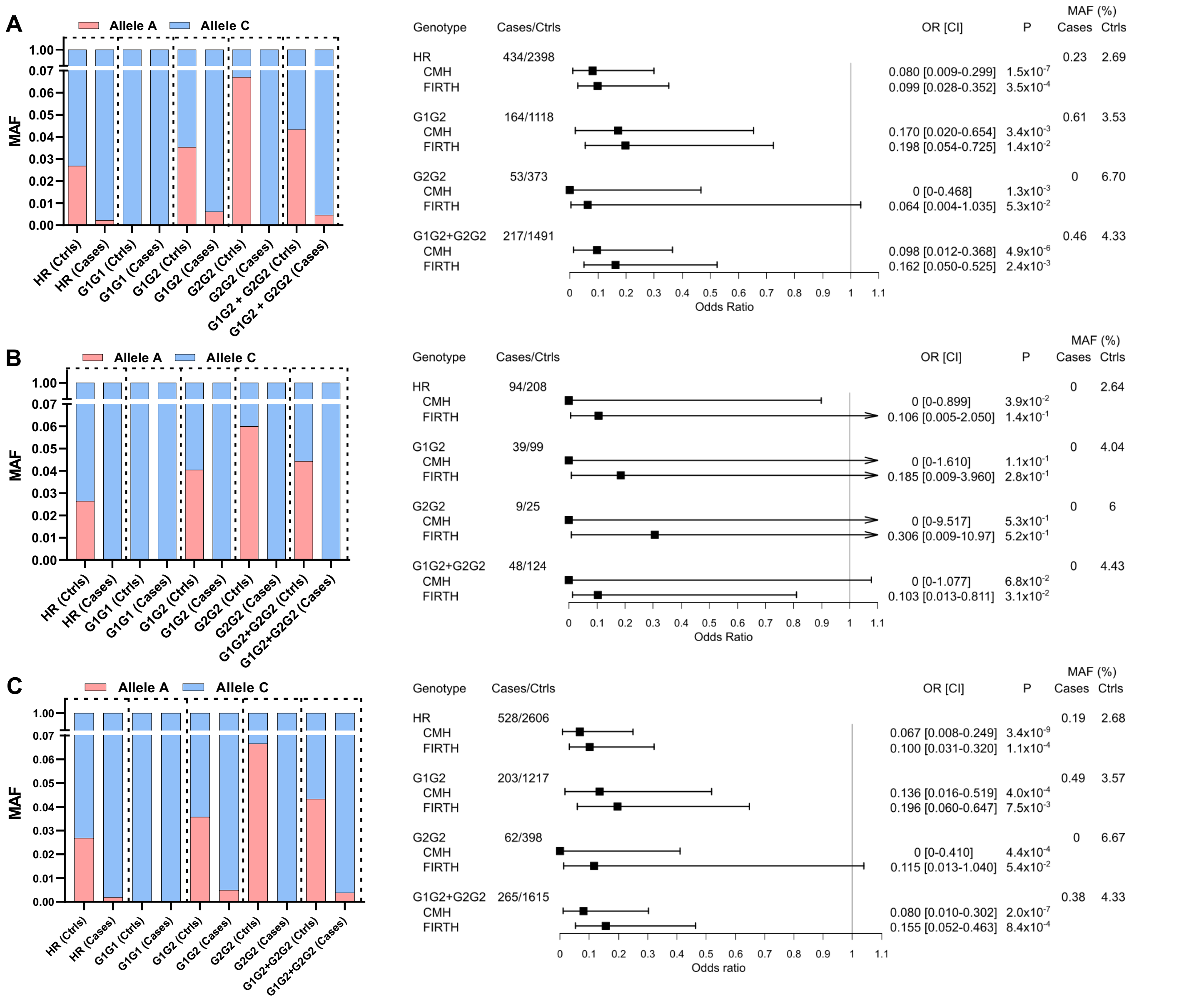

Stacked bar plots (left) and forest plots (right) for **A)** FSGS cohort 1, **B)** FSGS cohort 2 **C)** Meta-analysis of the pooled cohort. Forest plots with all cohorts' *APOL1* genotypes shown in the first column, and in the second column the number of cases and controls. In the third column odds ratios are shown (pooled for CMH and adjusted odds ratios for Firth Regression), with confidence intervals. *P* values are given in the 4th column, and minor allele frequencies (as percent) in cases and controls are listed in the last column. For every *APOL1* HR genotype the forest plot also provides summary statistics for both the CMH test and Firth Regression. CI=95% confidence interval.

Supplementary Figure 3. ***APOL1*** **p.N264K association with FSGS in EUR and AFR populations.**

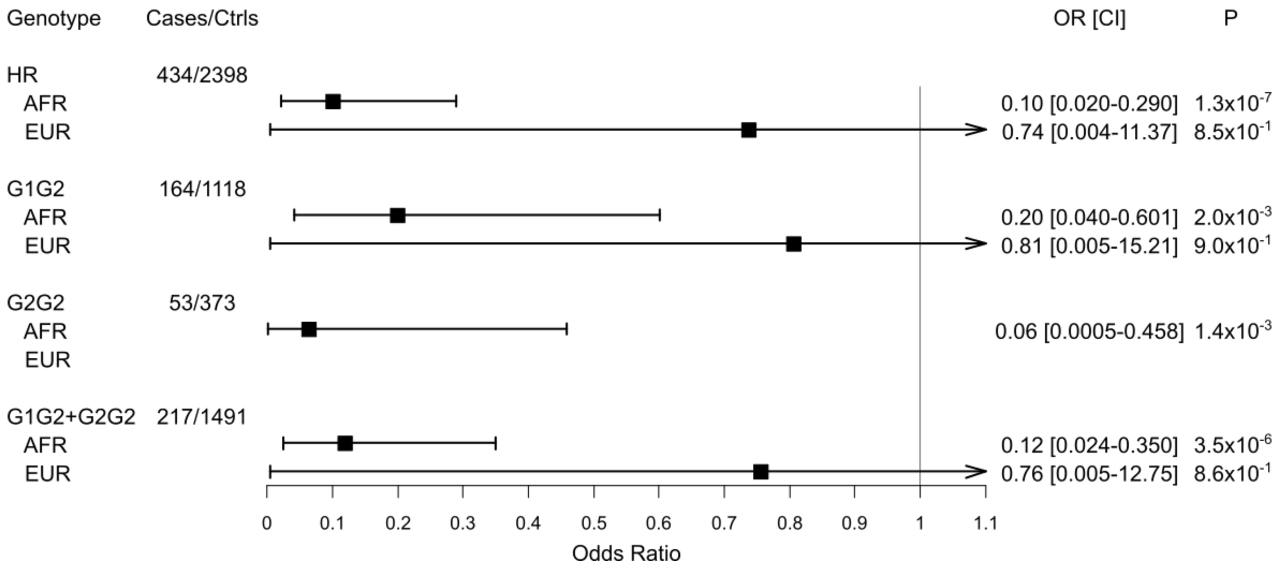

Forest plot summarizing local ancestry statistics using Tractor. Every group of rows shows different *APOL1-HR* genotypes and, in sub rows, the individual local ancestry (AFR, EUR) association after estimation with RFMix. The plot shows adjusted odds ratios and confidence intervals obtained using Firth regression after adjustment for sex and two principal components (PCs). The *P* values are provided in last column. CI=95% confidence interval.

Supplementary Figure 4. Principal component plots of *APOL1-HR* participants in the REGARDS and eMERGE-III Studies and forest plots of the association analyses in these cohorts.

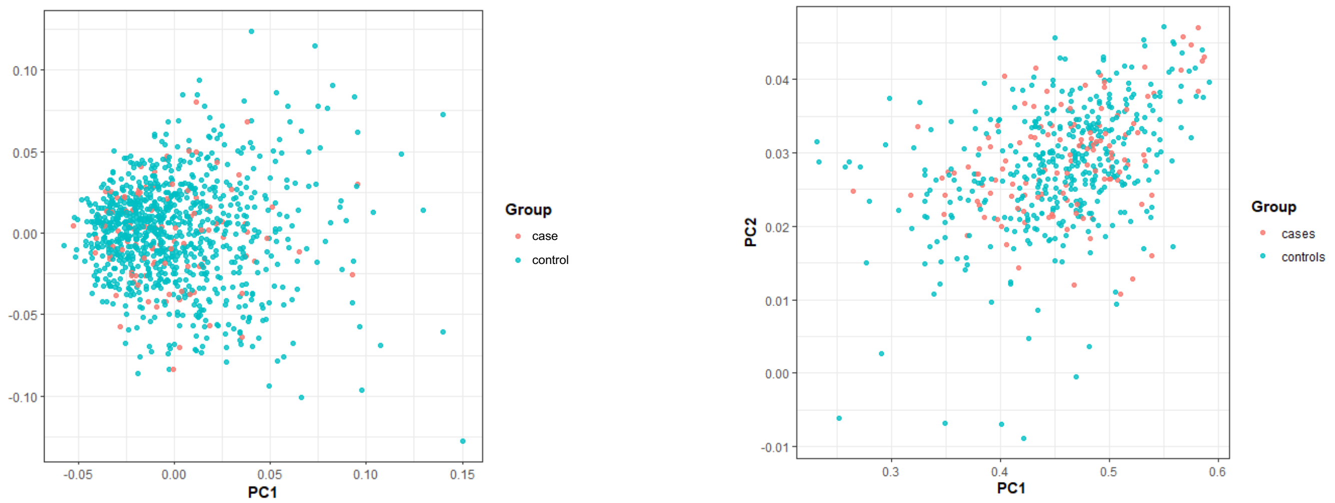

C.

Genotype	Cases/Ctrls	OR [CI]	P	MAF (%) Cases	MAF (%) Ctrls
HR					
REGARDS	150/893	0.261 [0.030-1.006]	0.055	0.67	2.58
EMERGE-III	126/404	0.332 [0.037-1.394]	0.192	0.79	2.35
JOINT REGARDS+EMERGE-III	276/1267	0.286 [0.103-0.792]	0.016	0.72	2.51
G1G2					
REGARDS	68/387	0.242 [0.006-1.517]	0.239	0.73	2.97
EMERGE-III	55/183	0.364 [0.008-2.681]	0.466	0.91	2.46
JOINT REGARDS+EMERGE-III	123/570	0.290 [0.033-1.155]	0.103	0.81	2.81
G2G2					
REGARDS	22/149	0.279 [0.007-1.810]	0.338	2.23	7.72
EMERGE-III	9/55	0.590 [0.013-4.660]	1.000	5.55	9.09
JOINT REGARDS+EMERGE-III	31/204	0.378 [0.043-1.544]	0.295	3.22	8.09
G1G2+G2G2					
REGARDS	80/536	0.251 [0.029-0.974]	0.036	1.11	4.29
EMERGE-III	64/238	0.382 [0.043-1.619]	0.276	1.56	3.99
JOINT REGARDS+EMERGE-III	144/774	0.300 [0.108-0.834]	0.023	1.29	4.19

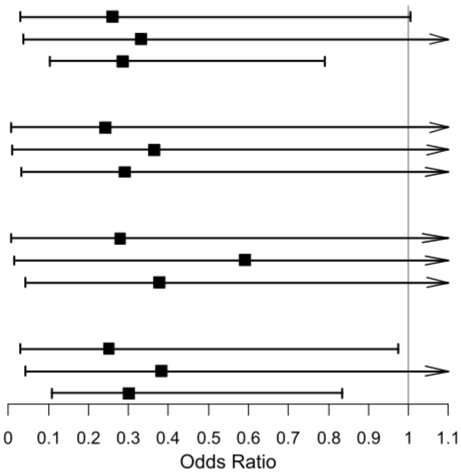

A-B. PCA plots for the *APOL1-HR* individuals from the REGARDS and EMERGE-III study used in the current analyses. **C.** Forest plots showing all cohorts with *APOL1* genotypes in the first column, number of cases and controls in the second column, odds ratios and confidence intervals in the third column (Fisher's exact test for REGARDS and eMERGE-III cohorts, and CMH test for the joint REGARDS+eMERGE-III analysis), followed by *P*-values, and minor allele frequency (MAF, expressed in percentage) in cases and controls. For every *APOL1* HR genotype the forest plot also provides summary statistics for both the CMH test and fisher test. CI=95% confidence interval. PC=principal component; HR=high risk; Ctrls=controls; OR=odds ratio; CI=confidence interval; MAF=minor allele frequency.

SUPPLEMENTARY TABLES

Supplementary Table 1. FSGS case-control discovery and replication cohorts

**FSGS cohort 1**		
**Recruitment Center**	**Number of cases**	**Genotyping Platform**
CUIMC	196	Illumina MEGA arrays
BIDMC	238	Illumina HumanOmniExpress-12
**Total**	**434**	
**FSGS cohort 2**		
**Recruitment Center**	**Number of cases**	**Genotyping Platform**
CureGN + Neptune + CUIMC	58	30X WGS
Duke University	36	30X WGS
**Total**	94	

Supplementary Table 2. Association of *APOL1* p.N264K to CKD-5 and ESKD in APOL1-HR individuals from the REGARDs and EMERGE-III studies.

**REGARDS**									
**APOL1 GT**	**MAF (%)**	**MAF CA (%)**	**MAF CO (%)**	**N. CA**	**N. CO**	**Fet OR**	**L5**	**U5**	**Fet P**
HR	2.30	0.67	2.58	150	893	0.261	0.030	1.006	0.055
G1G2	2.64	0.73	2.97	68	387	0.242	0.006	1.517	0.239
G2G2	7.02	2.23	7.72	22	149	0.279	0.007	1.810	0.338
G1G2+G2G2	3.83	1.11	4.29	80	536	0.251	0.029	0.0.974	0.036
**EMERGE-III**									
**APOL1 GT**	**MAF (%)**	**MAF CA (%)**	**MAF CO (%)**	**N. CA**	**N. CO**	**Fet OR**	**L5**	**U5**	**Fet P**
HR	1.98	0.79	2.35	126	404	0.332	0.037	1.394	0.192
G1G2	2.10	0.91	2.46	55	183	0.364	0.008	2.681	0.466
G2G2	8.59	5.55	9.09	9	55	0.590	0.013	4.660	1
G1G2+G2G2	3.48	1.56	3.99	64	238	0.382	0.043	1.619	0.276
**JOINT REGARDS – EMERGE-III**									
**APOL1 GT**	**MAF (%)**	**MAF CA (%)**	**MAF CO (%)**	**N. CA**	**N. CO**	**CMH OR**	**L5**	**U5**	**CMH P**
HR	2.19	0.72	2.51	276	1297	0.286	0.103	0.792	0.016
G1G2	2.44	0.81	2.81	123	570	0.290	0.033	1.155	0.103
G2G2	7.45	3.22	8.09	31	204	0.378	0.043	1.544	0.295
G1G2+G2G2	3.71	1.29	4.19	144	774	0.300	0.108	0.834	0.023

GT=genotype; MAF=minor allele frequency for the p.N264K variant; CA=cases; CO=controls; N.=number; Fet=Fisher's Exact Test; L5=lower 5% confidence interval; U5=upper 5% confidence interval.
