# Supplementary material for: Strong protective effect of the APOL1 p.N264K variant against G2-associated focal segmental glomerulosclerosis and kidney disease
Source: Nat Commun. 2023 Nov 30;14:7836. doi: 10.1038/s41467-023-43020-9 (PMC10689833; doi:10.1038/s41467-023-43020-9)
Supplement: Supplementary file 3 — Reporting Summary [file 41467_2023_43020_MOESM3_ESM.pdf]

nature portfolio

Corresponding author(s): Simone Sanna-Cherchi

Last updated by author(s): Oct 26, 2023

Reporting Summary

Nature Portfolio wishes to improve the reproducibility of the work that we publish. This form provides structure for consistency and transparency in reporting. For further information on Nature Portfolio policies, see our Editorial Policies and the Editorial Policy Checklist.

Statistics

For all statistical analyses, confirm that the following items are present in the figure legend, table legend, main text, or Methods section.

n/a	Confirmed	
☐	☒	The exact sample size (n) for each experimental group/condition, given as a discrete number and unit of measurement
☐	☒	A statement on whether measurements were taken from distinct samples or whether the same sample was measured repeatedly
☐	☒	The statistical test(s) used AND whether they are one- or two-sided<br>*Only common tests should be described solely by name; describe more complex techniques in the Methods section.*
☐	☒	A description of all covariates tested
☐	☒	A description of any assumptions or corrections, such as tests of normality and adjustment for multiple comparisons
☐	☒	A full description of the statistical parameters including central tendency (e.g. means) or other basic estimates (e.g. regression coefficient) AND variation (e.g. standard deviation) or associated estimates of uncertainty (e.g. confidence intervals)
☐	☒	For null hypothesis testing, the test statistic (e.g. F, t, r) with confidence intervals, effect sizes, degrees of freedom and P value noted<br>*Give P values as exact values whenever suitable.*
☒	☐	For Bayesian analysis, information on the choice of priors and Markov chain Monte Carlo settings
☐	☒	For hierarchical and complex designs, identification of the appropriate level for tests and full reporting of outcomes
☐	☒	Estimates of effect sizes (e.g. Cohen's d, Pearson's r), indicating how they were calculated

Our web collection on statistics for biologists contains articles on many of the points above.

Software and code

Policy information about availability of computer code

Data collection	NA
Data analysis	All details on data analysis are reported in the Supplementary Information Appendix. No commercial software was used. Open source publicly available softwares used: KING2.3.0; PLINK 2; GATK 4.3; rtracklayer R package; R 4.3; RFMix v2; logistf R package; FlashPCA; Tractor

For manuscripts utilizing custom algorithms or software that are central to the research but not yet described in published literature, software must be made available to editors and reviewers. We strongly encourage code deposition in a community repository (e.g. GitHub). See the Nature Portfolio guidelines for submitting code & software for further information.

Data

Policy information about availability of data

All manuscripts must include a data availability statement. This statement should provide the following information, where applicable:

- Accession codes, unique identifiers, or web links for publicly available datasets
- A description of any restrictions on data availability
- For clinical datasets or third party data, please ensure that the statement adheres to our policy

Illumina DNA microarray data generated for the FSGS cohort 1 analysis was used to extract genetic information for the p.N264K single variant analysis, associated only to a sparse markers map in order to generate principal components for ancestry adjustments. As such, a complete genome-wide analysis of these data has not

been yet conducted and the full data will be deposited in dbGaP at completion of the genome-wide analyses. These complete data are currently available to investigators upon reasonable request.

Research involving human participants, their data, or biological material

Policy information about studies with human participants or human data. See also policy information about sex, gender (identity/presentation), and sexual orientation and race, ethnicity and racism.

Reporting on sex and gender	We used only genetically-determined sex
Reporting on race, ethnicity, or other socially relevant groupings	We used only genetically determined ancestry
Population characteristics	Significant covariates: genetically-determined sex and genetic ancestry calculated via principal component analysis
Recruitment	Individuals considered as cases were enrolled via presence of a kidney biopsy diagnosis of focal segmental glomerulosclerosis (FSGS).
Ethics oversight	The IRB at Columbia University and Collaborating Institutions approved the study

Note that full information on the approval of the study protocol must also be provided in the manuscript.

Field-specific reporting

Please select the one below that is the best fit for your research. If you are not sure, read the appropriate sections before making your selection.

☒ Life sciences ☐ Behavioural & social sciences ☐ Ecological, evolutionary & environmental sciences

For a reference copy of the document with all sections, see nature.com/documents/nr-reporting-summary-flat.pdf

Life sciences study design

All studies must disclose on these points even when the disclosure is negative.

Sample size	The was no power calculation conducted to pre-determin sample size. This study leverages on a rare condition, focal segmental glomerulosclerosis (FSGS), which has a prevalence of 7 in a million. Our cohort represent the recruitment effort of multiple investigators over decades to accrue among the largest datasets reported thus far for this condition.
Data exclusions	We included in this study only individual of recent African ancestry (genetically determined) and, among those, only individuals who carried the APOL1 high-risk genotypes. This was done to assess the specific role on the APOL1 p.N264K missense variant in modifying the effect of APOL1 high-risk genotypes in affected and non affected individuals.
Replication	Replication studies were conducted in two large population-based cohort studies: the eMERGE Consortium and the REGARDS Study.
Randomization	This is a genetic case-control study, and established co-variates were used in all analyses, including sex and genetic ancestry (by use of principal component analysis).
Blinding	There is not blinding process in genetic association studies except that clinical recruiters who enroll patients for genetic studies are unaware of the analyses, and computational biologists who conduct the genetic associations are not involved in the phenotype adjudication.

Reporting for specific materials, systems and methods

We require information from authors about some types of materials, experimental systems and methods used in many studies. Here, indicate whether each material, system or method listed is relevant to your study. If you are not sure if a list item applies to your research, read the appropriate section before selecting a response.

Materials & experimental systems

n/a	Involved in the study
☒	☐ Antibodies
☒	☐ Eukaryotic cell lines
☒	☐ Palaeontology and archaeology
☒	☐ Animals and other organisms
☒	☐ Clinical data
☒	☐ Dual use research of concern
☒	☐ Plants

Methods

n/a	Involved in the study
☒	☐ ChIP-seq
☒	☐ Flow cytometry
☒	☐ MRI-based neuroimaging
